# FoCUS cardiac ultrasound training for undergraduates based on current national guidelines: a prospective, controlled, single-center study on transferability

**DOI:** 10.1186/s12909-023-04062-1

**Published:** 2023-02-01

**Authors:** Johannes Weimer, Peter Rolef, Lukas Müller, Henrik Bellhäuser, Sebastian Göbel, Holger Buggenhagen, Andreas Weimer, Elias Waezsada, Friederike Kirchhoff, Julia Weinmann-Menke

**Affiliations:** 1 Rudolf-Frey Lernklinik, Department of Medicine, University of Mainz, Langenbeckstraße 1, 55131 Mainz, Germany; 2 Department of Diagnostic and Interventional Radiology, University of Mainz, Mainz, Germany; 3 Institute of Psychology, Johannes Gutenberg University of Mainz, Mainz, Germany; 4 Department of Medicine II, Cardiology Center, Department of Medicine, University of Mainz, Mainz, Germany; 5 Center for Orthopedics, Emergency Surgery, and Paraplegics, Department of Medicine, University of Heidelberg, Heidelberg, Germany; 6 Department of Cardiology, Kerckhoff Hospital, Bad Nauheim, Germany; 7 Department of Medicine I, Nephrology Center, Department of Medicine, University of Mainz, Mainz, Germany

**Keywords:** Ultrasound education, Echocardiography, Focused cardiac ultrasound, Peer assisted learning, Curriculum development

## Abstract

**Introduction:**

In emergency and critical-care medicine, focused cardiac ultrasound (FoCUS) is indispensable for assessing a patient’s cardiac status. The aim of this study was to establish and validate a peer-to-peer–supported ultrasound course for learning FoCUS-specific skills during undergraduate studies at a German university.

**Methods:**

A 1-day, 12 teaching units training course was developed for students in the clinical section of medical college, with content based on the current national guidelines. A total of 217 students participated in the study (97 in the course group and 120 in the control group). The course and the participants’ subjective assessment of improved skills were evaluated using a questionnaire (7-point Likert scale; 7 = complete agreement and 1 = no agreement at all). Objective learning gains were assessed by tests before and after the course. These consisted of a test of figural intelligence (eight items) and a test of technical knowledge (13 items).

**Results:**

The course participants experienced significant improvement (*P* < 0.001) from before to after the course, with a large effect size of *η*^*2*^_*part*_ = 0.26. In addition, the course group had significantly better results (*P* < 0.001) than the control group in the post-test, with a medium to large effect size of *η*^*2*^_*part*_ = 0.14. No significant differences (*P* = 0.27) were detected in the test section on figural intelligence. The evaluations showed that the participants had a high degree of satisfaction with the course approach, teaching materials, and tutors. There was also a positive increase in their subjective assessment of their own skills, including areas such as technical knowledge, ultrasound anatomy, and performance of the examination.

**Conclusion:**

The results of both the objective learning assessment and the subjective evaluations suggest that a FoCUS course originally intended for qualified physicians is equally suitable for students. With the development and provision of modern digital teaching media, even more students will be able to benefit from this approach in the future.

**Supplementary Information:**

The online version contains supplementary material available at 10.1186/s12909-023-04062-1.

## Introduction

### Importance of echocardiography and examination concepts

Echocardiography is one of the most important pillars of cardiological diagnosis [1]. It is the most readily available and most commonly used machine-aided, gold-standard diagnostic technique for the cardiovascular system [2–5]. Thanks in particular to the introduction of more mobile and powerful devices, the examination method is increasingly being used in a more flexible and location-independent manner [6–14].

The terms “clinical echocardiography” [2], “emergency echocardiography” [15], and “focused cardiac ultrasound” (FoCUS) [10, 11] have become established to refer to ultrasound examinations of the heart. These reflect different approaches to the examination and different specialties [2, 10, 11]. The complex and technically demanding technique of clinical echocardiography is usually performed by cardiologists [2]. By contrast, FoCUS plays a more important role in initial assessment and follow-up by noncardiological specialists in other areas such as emergency and intensive-care medicine [12]. For this purpose, algorithms for FoCUS have been developed to enable physicians to carry out focused examinations after short training periods [11–13, 15].

### Training approaches for physicians and students

Medical training in echocardiography is structured in very different ways internationally. Various national and international specialist societies provide guidelines and recommendations for medical training in echocardiography [12, 15–17]. The content recommended by these institutions is taught in continuing education courses that reflect different examination systems and levels of competence [18]. FoCUS courses are in strong demand, and participation in these certified courses is now part of the further training curriculum in anesthesiology and intensive care at many German hospitals [19].

Teaching and learning practical cardiac-specific ultrasound skills in the context of student (pre)clinical training has been the subject of recent scientific investigations [20–31]. However, the educational approaches published so far vary widely, [32, 33]. In the future practical skills are likely to play a greater role in the clinical section of medical training, that is why it appears essential to establish a well-founded training approach for FoCUS courses even in the early period of medical training [34, 35].

### Research issue and aim of the present study

Our own preliminary research shows that there is considerable interest in echocardiography among students and that students think that practical skills in particular are not adequately covered in the curriculum [36]. In addition, it is becoming increasingly clear, in view of the National Catalogue of Competence-Based Learning Objectives in Medicine (*Nationaler Kompetenzbasierter Lernzielkatalog der Medizin,* NKLM), that advanced clinical skills such as echocardiography will also be included in the curriculum at German universities in the future [35]. The aim of the present study was therefore to evaluate the extent to which an existing recognized, certified course design originally intended for physicians [37] is capable of being transferred to students and established for this purpose after any necessary adaptations.

## Materials and methodology

### Course development and implementation

To answer the above question, a FoCUS-specific course approach was developed in 2018 at Mainz University Medical Center by the “sonoforklinik-students” working group, which at that time had already successfully established several training approaches in the field of abdominal ultrasonography [36]. For this purpose, an approach for perioperative focused echocardiography (PFE), designed in accordance with the guidelines of the German Society for Anesthesiology and Intensive-Care Medicine (*Deutsche Gesellschaft für Anästhesiologie und Intensivmedizin,* DGAI) was adjusted and adapted [37, 38]. This is a recognized training approach that is already being implemented at many hospitals in the continuing education of anesthesiologists, as well as intensive-care and emergency medicine physicians [19]. This approach is based on the sectional planes in transthoracic echocardiography proposed by the World Interactive Network Focused on Critical Ultrasound (WINFOCUS) [38]. The project had interdisciplinary support and was monitored by partners from various departments at Mainz University Medical Center (internal medicine, emergency medicine, cardiology, anesthesiology, radiology, teaching hospital/education) and external institutions (Heidelberg University Hospital, Kerckhoff Hospital in Bad Nauheim).

**Fig. 1:**
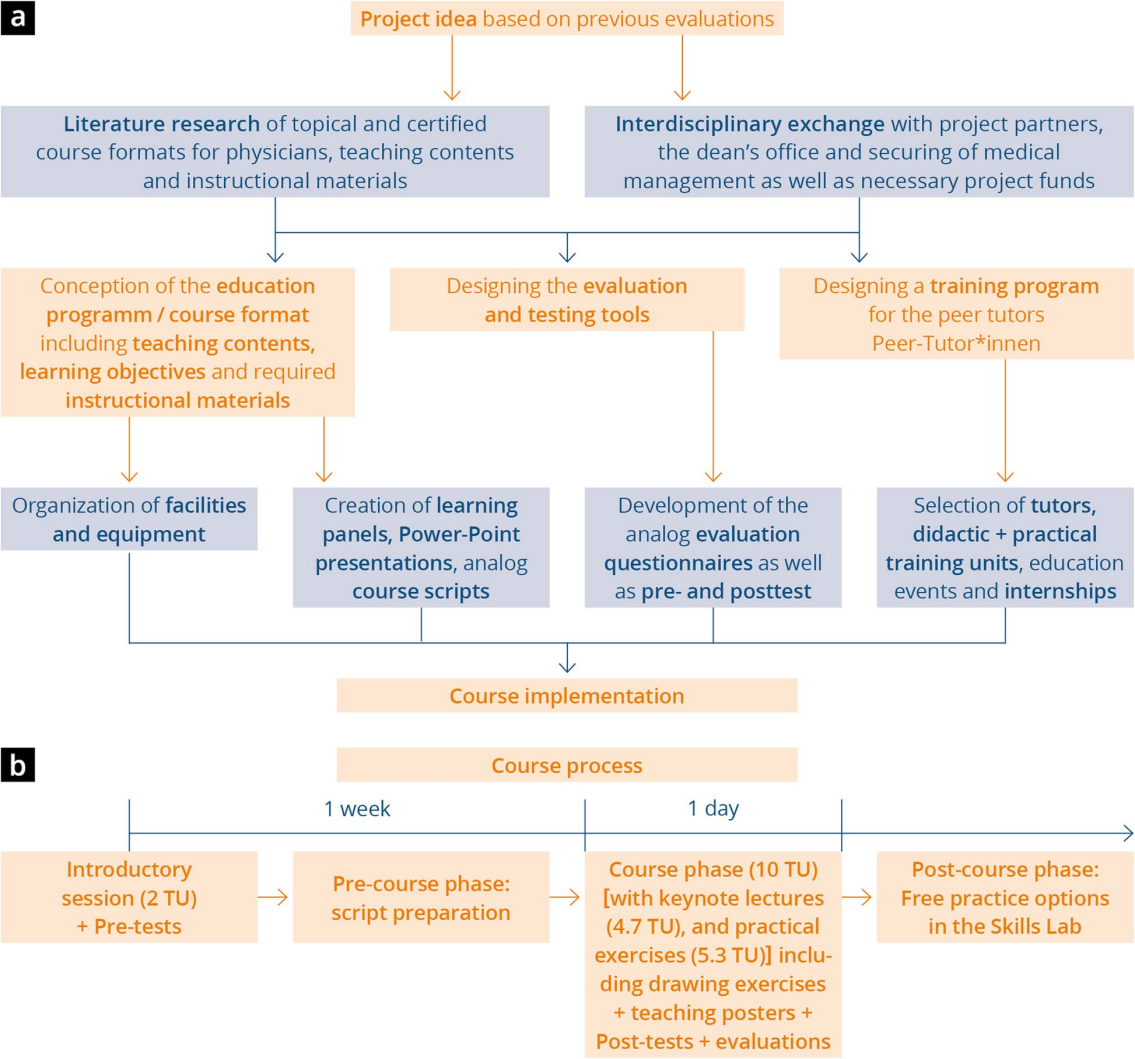
Process of course development, from the idea to the implemented project, including the course sequence with each teaching unit (TU, teaching units)

The training approach, which comprises a total of 12 teaching units (TUs), includes an introductory session (2 TUs), theory units with keynote lectures (4.7 TUs), and practical exercises (5.3 TUs). The learning objectives defined and the modified course structure and sequence developed are shown in Table 1, Fig. 1, Supplements 1 and 2. In the introductory session, which was held 1 week before the course, the participants were provided with general information, a theoretical introduction to the equipment, and basic information about the image acquisition process. In addition, pre-course tests were conducted, including requesting the participants’ demographic data, and the teaching material (lecture notes) was handed out. The students did not receive any feedback on the pre-course test. The theory units and tests on the day of the course were held in plenary sessions. In the practical units, one tutor taught a maximum of five students, who carried out ultrasound examinations on each other. For each topic, the students changed rooms and tutors. At the end of the course, a post-course test and evaluation were conducted and the participants each received an educational wall poster for memorizing content and follow-up on the course. Following the course, there was an optional opportunity to attend free practice slots without an instructor in Mainz University Medical Center’s Skills Lab.

**Table 1:** Learning objectives in the FoCUS course model developed and adapted from Greim et al. [37]

Topic	Learning objectives: on completing the course, the participant should be able to:
Basic anatomy and physiology	— Describe anatomic relationships in the chest
	— Show the exact position of the heart and the positional relationship of the chambers to each other
	— Explain the physiology of blood flow and valve mechanics during systole and diastole
Physical principles, choice of transducer, and instrument buttons	— Explain the physical principles of image acquisition and ultrasound diagnosis
	— Explain the differences between the various transducers and justify the choice of transducer
	— Explain how penetration depth, image resolution, and frequency are related to each other
Patient positioning and transducer movement	— Carry out positioning variations and explain how positioning improves the ultrasound conditions
	— Understand and implement defined movement sequences (tilting, angulating, rotating, shifting, swiveling) in relation to image morphology
Examination	— Adjust the defined standard sections (PLAX; PSAX; A4K, SIVC; S4K) and explain the plane
	— List the possible questions that can be answered by setting the respective plane
	— Describe the possible transducer movements and buttons available to optimize the planes

### Tutors (and tutor training)

A total of six student peer tutors recruited from semesters 8 to 10 were trained in a multistage process that included participation in a German Society of Ultrasound in Medicine (DEGUM (Deutsche Gesellschaft für Ultraschall in der Medizin)) or DGAI-certified ultrasound course, internal technical and educational training (30 TU), and job shadowing in the ultrasound laboratory (with performance of at least 50 independent examinations). The acquisition of competence through this multistage training program was verified using a practical examination which the physicians conducting this study executed. The peer tutors had to demonstrate all the practical, theoretical and didactic skills required for the course in this trial session.

### Premises and equipment

The course and seminar rooms of the Rudolf Frey Teaching Hospital were used for the practical and theoretical units. Five ultrasound systems from Phillips (CX 50, ClearVue 550) and General Electric (GE F8) were used, equipped with sector probes and presets suitable for echocardiography.

### Teaching and learning materials

The development and design of the learning materials (lecture notes and educational wall poster), as well as teaching materials (PowerPoint presentations), were carried out by the tutors in collaboration with the project partners and with the help of the technical literature. The integrated learning software in the GE-F8 devices and the “TTE FOCUS Views” app were also included in the hands-on ultrasound course [39, 40].

### Recruitment and sampling

A total of 97 students received training in six courses between the winter semester of 2018–19 and the winter semester of 2019–20. The intervention group consisted of students from all clinical semesters of medical school (semester 5–10), who were assigned to the course after prior voluntary registration via the “Ilias” platform. Four of the participants were not included in the study due to incomplete test forms. The control group consisted of voluntary participants (*n* = 120) from the first clinical semester (semester 5). They were currently attending their internal medicine examination course during the summer semester of 2020, and only completed the pre-course test and the post-course test 1 week later. This group received no ultrasound training between the two tests. The demographic data for the two groups *prior to the initial test (pre-course test)* are shown in Table 2.

**Table 2:** Baseline characteristics of the study participant groups

	Course group (*n* = 93)		Control group (*n* = 120)	
Gender (n, %)	F 54 (58)	M 39 (42)	F 64 (53)	M 56 (47)
Age				
Mean (SD)	25 (3.6)		26 (3.5)	
Median	24		25	
	Yes	No	Yes	No
Experience and knowledge prior to medical school				
Studies in other subjects than medicine (n, %)	59 (63)	34 (37)	90 (75)	30 (25)
Prior knowledge of medicine (n, %)	56 (60)	37 (40)	83 (69)	37 (31)
Participation of the official study aptitude test medicine (n, %)	45 (48)	48 (52)	50 (42)	70 (58)
Experience and knowledge prior to the pre-test (during medical school)				
Prior knowledge of ultrasound (n, %)	80 (86)	13 (14)	67 (56)	53 (44)
Prior knowledge of echocardiography (n, %)	13 (14)	80 (86)	4 (3)	116 (97)
Any other ultrasound course completed (n, (%)	63 (68)	30 (32)	56 (47)	64 (53)
Any other echocardiography course (n, %)	10 (11)	83 (89)	0 (0)	120 (100)

### Test and evaluation instruments

The data were collected from the pre-course and post-course tests and evaluations conducted as part of the quality assurance process for the course. Data from a total of 93 theory tests and 97 evaluations were collected. The tests were assigned using the course number (1–6) and sequential numbers assigned before the pre-course test. No tests were directly linked to the evaluation questionnaires.

The test questionnaires (for an excerpt, see Supplement 3) comprised a total of eight multiple-choice questions on “figural intelligence” in the form of tubular figures and 13 questions on “technical knowledge”. The questions in the tests were shown to the participants via a PowerPoint presentation in plenary sessions. The participants had 45 seconds each to answer the questions on “figural intelligence” and 1.5 min each to answer the questions on “technical knowledge”. The test was administered to the control group with the same time constraints. In addition, the students completed drawing exercises on the sectional planes listed in the learning objectives before, during, and after the course. The evaluation of these was not included in the final assessment.

In addition to questions on demographic data as part of the pre-course test, inquiries were made about nine subject complexes (“expectations and needs”, “course and course structure”, “learning methods, media and materials”, “skills before completing the course”, “skills after completing the course”, “skills development relative to further learning objectives”, “tutors’ skills”, “tutors’ presentation and educational skills” and “use of digital teaching media in ultrasound teaching”) using a total of 62 items based on a seven-point Likert scale (“7” = complete agreement and “1” = no agreement at all).

### Study design and statistical analysis

Data for this prospective and controlled single-center study were collected using Microsoft Excel and analyzed using IBM SPSS version 27.0.1.0 (IBM Corporation, Armonk, New York). JASP (Jeffrey’s Amazing Statistics Program) version 0.16.2 (JASP Team, University of Amsterdam) was used to perform the statistical tests and create the graphs. All qualitative data from the evaluation form are presented in purely descriptive terms and are not therefore subject to significance testing. The quantitative data from the test on successful learning were compared using two-factor repeated-measures ANOVA (significance level *P* = 0.05). The main focus was on evaluating the interaction effect of the intervention — i.e., whether the intervention group experienced greater improvement between the pre-course and post-course test than the control group.

## Results

### Test results

**Fig. 2:**
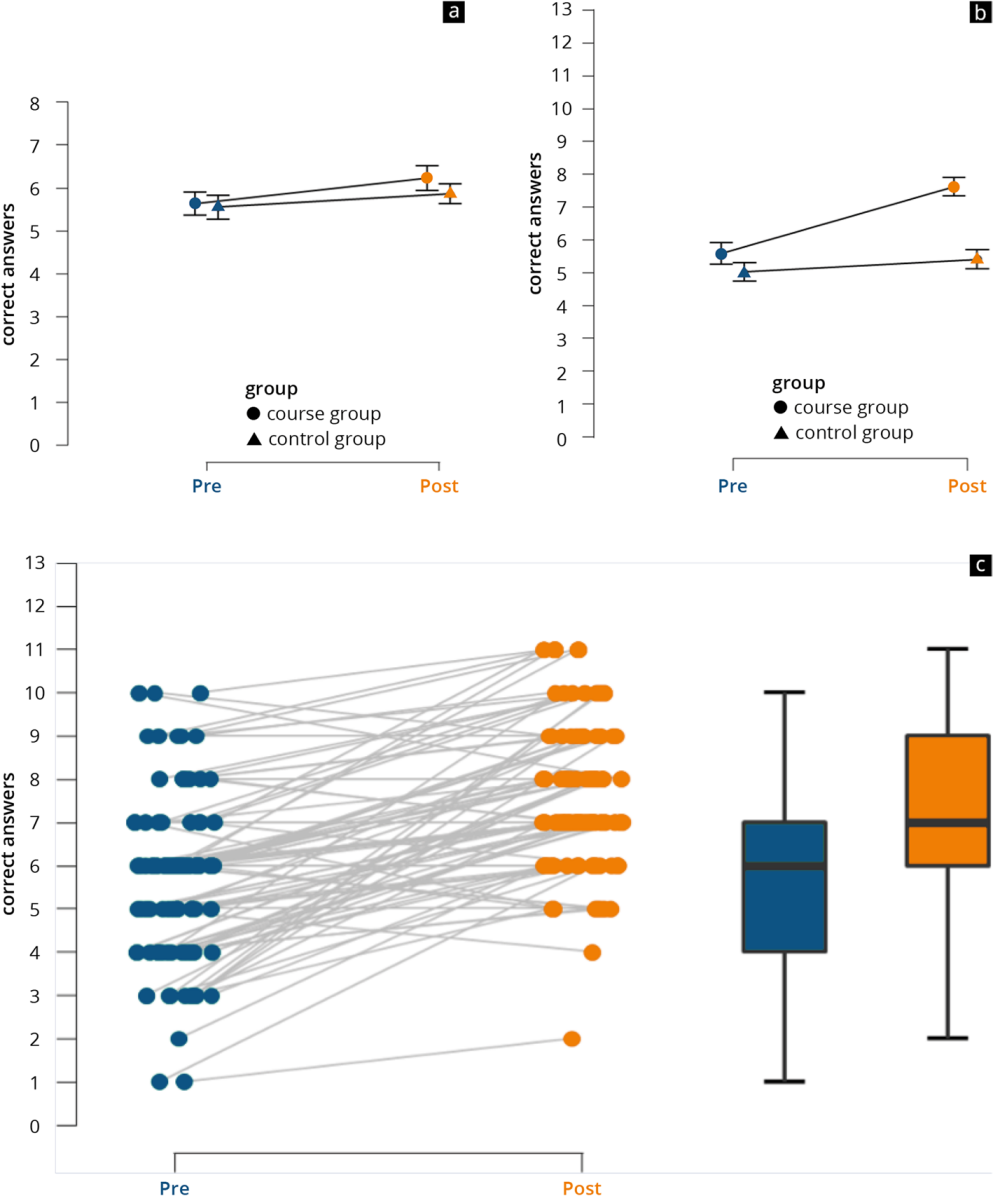
Results of the test sections on “figural intelligence” (**a**) and “technical knowledge” (**b**) in the pre-course and post-course tests and analysis of individual progress (**c**)

Figure 2a shows the results of Test 1, on “figural intelligence”. In this test, the control group improved by a mean of 0.4 points, from 5.5 (SD 1.8) to 5.9 (SD 1.6), and the course group improved by 0.6 points from 5.6 (SD 1.7) to 6.2 (SD 1.6). Time as the main effect was significant in the test, with a *P* value of < 0.001 and a mean effect size of *η*^*2*^_*part*_ = 0.06. This implies that both groups show mean improvement between the pre-course and post-course tests. Group alone as the main effect (*P* = 0.27) proved not to be significant in relation to the tubular figures. The interaction effect was also not significant (*P* = 0.26).

Figure 2b presents the results of Test 2 for the subject complex of “technical knowledge”. Here again, the control group improved by a mean of 0.4 points from 5 points (SD 1.8) to 5.4 points (SD 1.6). By contrast, the course group increased by 2 points, from 5.6 (SD 1.9) to 7.6 (SD 1.8). Time as the main factor also reached significance (*P* < 0.001) in this part of the test, with a large effect size of *η*^*2*^_*part*_ = 0.26. The group as main effect alone reached significance (*P* < 0.001), with a large effect size of *η*^*2*^_*part*_ = 0.19. The same was also the case with the interaction effect, which reached significance in test 2, with a medium to large effect size of *η*^*2*^_*part*_ = 0.14 (*P* < 0.001).

Figure 2c indicates the growth in individual skills in the course group. It can be seen that the majority of the participants were able to improve individually. Particularly in the lower score ranges (1–6 points), an increase is also evident from the box plots, with the median increasing from six to seven points. In addition, both the first and the third quartiles increased by two points each. The maximum and minimum scores also improved by one point each.

### Evaluation results

Table 3 presents the results of the evaluation that was carried out and the subjective competence assessment. Overall, there was a high level of satisfaction among the course participants in all of the items surveyed (mean > 6), as well as an increase in the competence assessment by at least 2 points on the scale, with the largest increase in “conducting a FoCUS examination”.

**Table 3:** Course participants’ evaluation results for the questionnaire items

**Course and course structure, teaching and learning materials**		**Expectations of the course**				**Approach/ outline**				**Achievement of learning objectives**				**Teaching materials**				**PowerPoints**			
**n**		97				97				97				96				97			
**Mean (SD)**		6.67 (0.52)				6.49 (0.56)				6.53 (0.65)				6.40 (0.86)				6.26 (1.0)			
**Min.**	**Max.**	3		7		5		7		4		7		3		7		1		7	
**Tutor expertise, practical/educational**		**expertise**				**Organ imaging**				**Presentation**				**Communication skills**				**Teaching**			
**n**		97				97				96				96				96			
**Mean (SD)**		6.41 (0.59)				6.72 (0.47)				6.7 (0.56)				6.69 (0.51)				6.52 (0.75)			
**Min.**	**Max.**	4		7		4		7		4		7		4		7		3		7	
**Subjective competence assessment**		**expertise**				**Organ imaging**				**Ultrasound anatomy**				**Patient guidance**				**FoCUS performance**			
		*Pre*		*Post*		*Pre*		*Post*		*Pre*		*Post*		*Pre*		*Post*		*Pre*		*Post*	
**n**		96		96		96		97		97		97		97		97		95		95	
**Mean (SD)**		3.81 (1.66)		5.64 (0.89)		4.07 (1.56)		5.8 (0.90)		4.21 (1.46)		5.88 (0.86)		4.67 (1.66)		5.99 (0.90)		2.11 (1.61)		4.65 (1.39)	
**Min.**	**Max.**	1	7	3	7	1	7	3	7	1	7	3	7	1	7	3	7	1	7	1	7

## Discussion

This study shows that a certified FoCUS course approach originally intended for physicians can be successfully transferred to students after suitable modification with the assistance of student peer tutors and be implemented and established on a lasting basis.

Transferring this type of approach to student training might in the future be able to counteract the wide range of different approaches to ultrasound training described in the literature, by providing a more standardized structure [32, 41, 42]. However, an absolute prerequisite for this would be involvement and organization by the recognized specialist societies, including exchanges of expert information on educational approaches. Even today, more and more emphasis is being placed on practical training in the clinical section of medical courses, with certain levels of skill being required before an individual begins to practice medicine. The easier transference to clinical specialist training resulting from this would be another advantage. This development is also in line with the goals of the NKLM [34] and the curricula at German universities.

However, providing the staffing resources required for planning and implementing a FoCUS course may prove difficult for teaching institutions. It is therefore worthwhile to try to incorporate well-established and certified course models that already exist into the curriculum [41]. In the future, such courses should be centrally administered at universities and organized and structured with the help of teaching/learning workshops in order to conserve resources and reduce the burden on departments.

It is already known that students have a positive attitude to practical formats for ultrasound training [20, 25, 43–45]. Our data also reflect these findings and show that it is useful and desirable to integrate the teaching of echocardiographic skills into student training curricula, with particular emphasis on combining theoretical knowledge with practical content. To achieve this, greater emphasis should and must be placed on training tutors, developing and producing innovative teaching materials, and on the internal structures that are important for implementing such courses [46–48].

To make it possible to offer practical courses to as many students as possible, it is useful and essential to use peer-group tutors [21, 22, 49]. Since practical ultrasound courses work best in small groups [20] and are very time-consuming and resource-intensive, well-trained tutors could help to relieve the teaching loads of physicians and reduce group size. As in the preliminary study, the evaluation results presented here confirm and emphasize the participants’ degree of satisfaction with student tutors. The positive feedback from the participants in relation to the tutors’ ability to communicate, their presentation, and their teaching skills also shows that structured, qualitative training is feasible and indispensable [50, 51]. Since very few of the studies published to date that also use peer-supported training models have discussed the training process in greater detail [21, 22, 26, 27], a national and international standard needs to be established in this area in the future. This could lead to basic levels of competence among student tutors that would be equivalent to those of experts [52], ensuring even better quality in the training formats.

The form of preparation using lecture notes described in this training approach, which along with the PowerPoint slides used was positively evaluated by the participants, represents a fundamental element in achieving the best possible improvement in skills [53]. Attention also needs to be given here to the visual design, which influences students’ motivation to read the notes [53, 54]. Alongside the use of analogue learning materials [28], there is also a clear trend toward increasing digitalization in ultrasound training [23, 29, 30, 55, 56]. The development and use of e-learning tools [24] should be intensified in the future so that echocardiographic skills can be taught using a “blended learning” approach [32, 57] — a view that is also supported by some of the text comments provided in the evaluations. Since information about pathological findings was only conveyed in theoretical form in the present approach, the use of ultrasound simulators might allow a more practically oriented form of pathology training that would not have to take place directly on the patient [23, 31, 58]. Important factors that would have to be considered here include realistic implementation and haptics, as well as the usually high acquisition costs. In addition to the console equipment used in this study, an optimized training curriculum also ought to include the use of pocket devices [14, 59, 60].

In general, written tests [20, 22, 24, 25, 30], practical examination formats [20, 61], and self-assessments using questionnaires [61] are used to test improvements in skills. The test section on “figural intelligence” was intended to investigate whether the groups were comparable in relation to spatial orientation. The results support this assumption, although — contrary to expectations — both groups achieved significantly better results in the pre-course to post-course tests. It cannot be assumed that the participants acquired better spatial thinking abilities during the course; instead, it is more likely that they became more familiar with the questions and the short time frame available for answering them.

The data obtained in the second test show a significant increase in technical knowledge in the course group in comparison with the control group, a finding that is also consistent with the results for subjective competence assessments in the course group. Earlier studies also demonstrated this, although in the present study the training time and training formats were very different [22–27, 30]. A possible extension of the present training curriculum, which was adapted from the DGAI guidelines [37], might be to use standardized digital tests as well as practical examination formats [61].

Limitations of the present study that should be mentioned are the fact that no pathological findings were included in the theoretical test (although they were mentioned in theory classes) and there was no follow up. In addition, the data were collected over several courses, and practical test formats were not used to measure skills. A bigger test group would also have been preferable. Matching of subjective and objective skills assessments was also not possible, as the questionnaire and test sheets were independent.

## Conclusions

This study shows that our focused cardiac ultrasonography course was well received by the participants and every aspect of it was positively evaluated. With the help of the tests and evaluations conducted, it was shown that even existing course approaches designed for practicing physicians can be successfully incorporated into the teaching of medical students, with the help of student peer tutors.

## Supplementary Information

**Additional file 1: Supplement 1.** Course sequence, adapted from Greim et al. [37]. **Supplement 2.** Rotation plan for the practice group. MAPSE, mitral annular plane systolic excursion; TAPSE, tricuspid annular plane systolic excursion; EPSS, mitral valve E-point to septal separation. **Supplement 3.** Excerpt from the test sections on “figural intelligence” and “technical knowledge”.

## Data Availability

Data cannot be shared publicly because of institutional and national data policy restrictions imposed by the Ethics committee since the data contain potentially identifying study participants’ information. Data are available upon request from the Johannes Gutenberg University Mainz Medical Center (contact via weimer@uni-mainz.de) for researchers who meet the criteria for access to confidential data (please provide the manuscript title with your enquiry).
