# Supplementary material for: FoCUS cardiac ultrasound training for undergraduates based on current national guidelines: a prospective, controlled, single-center study on transferability
Source: BMC Med Educ. 2023 Feb 1;23:80. doi: 10.1186/s12909-023-04062-1 (PMC9893662; doi:10.1186/s12909-023-04062-1)

**Supplement 1.** Course sequence, adapted from Greim et al. [37]

| 9.00–9.30 a.m. | Welcome and presentation of the program, including learning goals |
| --- | --- |
| 9.30–10.00 a.m. | Technical, anatomic, and functional basics |
| 10.00–10.30 a.m. | Standard sections and settings options |
| 10.30–11.00 a.m. | Live ultrsound and knobology |
| 11.00 a.m.–1 p.m. | Practice cycle 1 |
| 1.00–1.30 p.m. | Lunch break |
| 1.30– | Right ventricular and left ventricular function (overview) |
| –2.15 p.m. | Pathologies + use of ultrasound in resuscitation |
| 2.15–2.30 p.m. | Break |
| 2.30–4.30 p.m. | Practice cycle 2 |
| 4.30–4.45 p.m. | Break |
| 4.45–5.30 p.m. | Documentation with quiz |
| 5.30 p.m. | End of course, farewell, certificates |
| Practical time: 240 min = 5.33 teaching units; theory: 210 min = 4,66 teaching units | |

**Supplement 2.**Rotation plan for the practice group. MAPSE, mitral annular plane systolic excursion; TAPSE, tricuspid annular plane systolic excursion; EPSS, mitral valve E-point to septal separation.

*
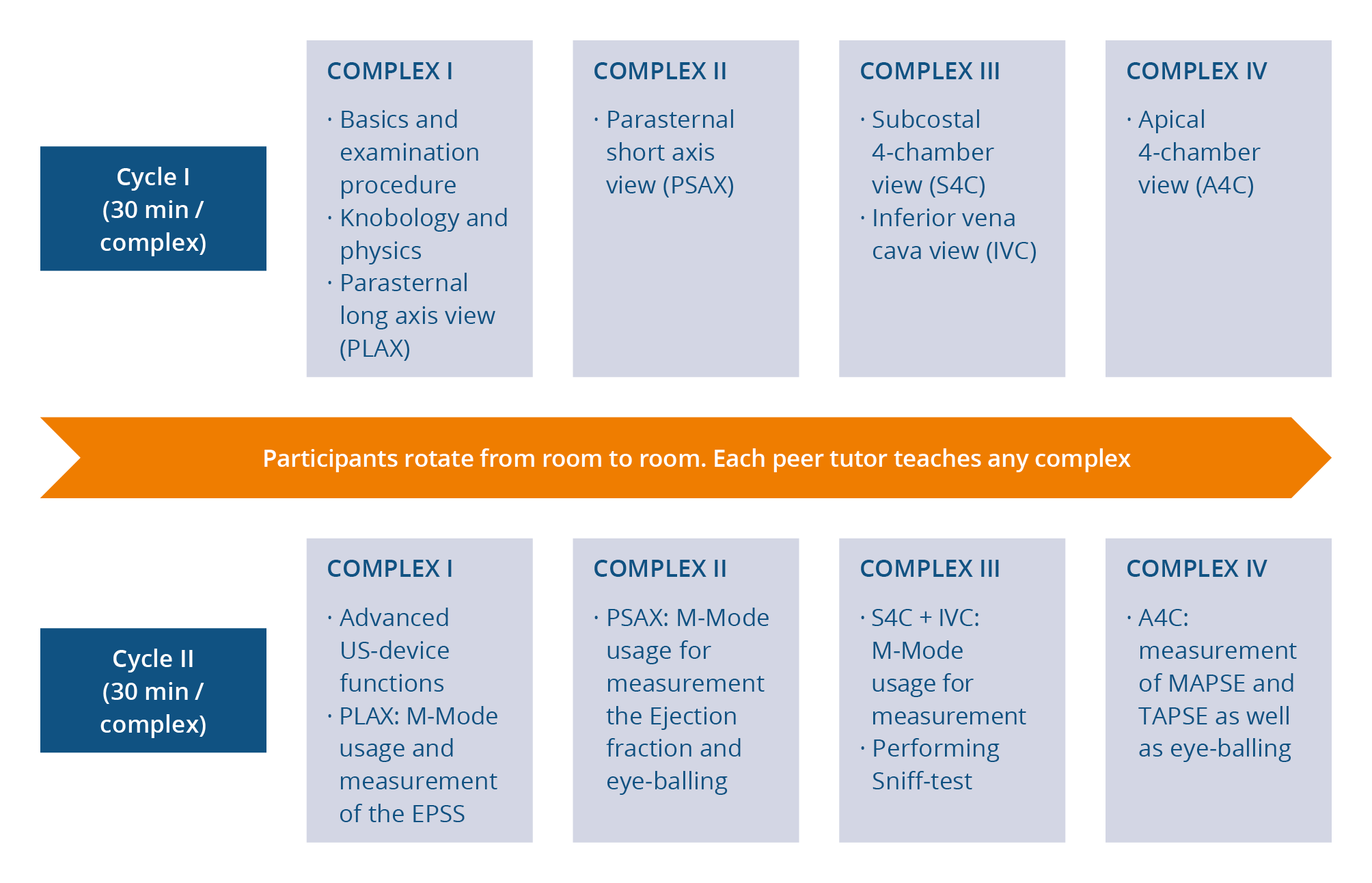
*

**Supplement 3.** Excerpt from the test sections on “figural intelligence” and “technical knowledge.”


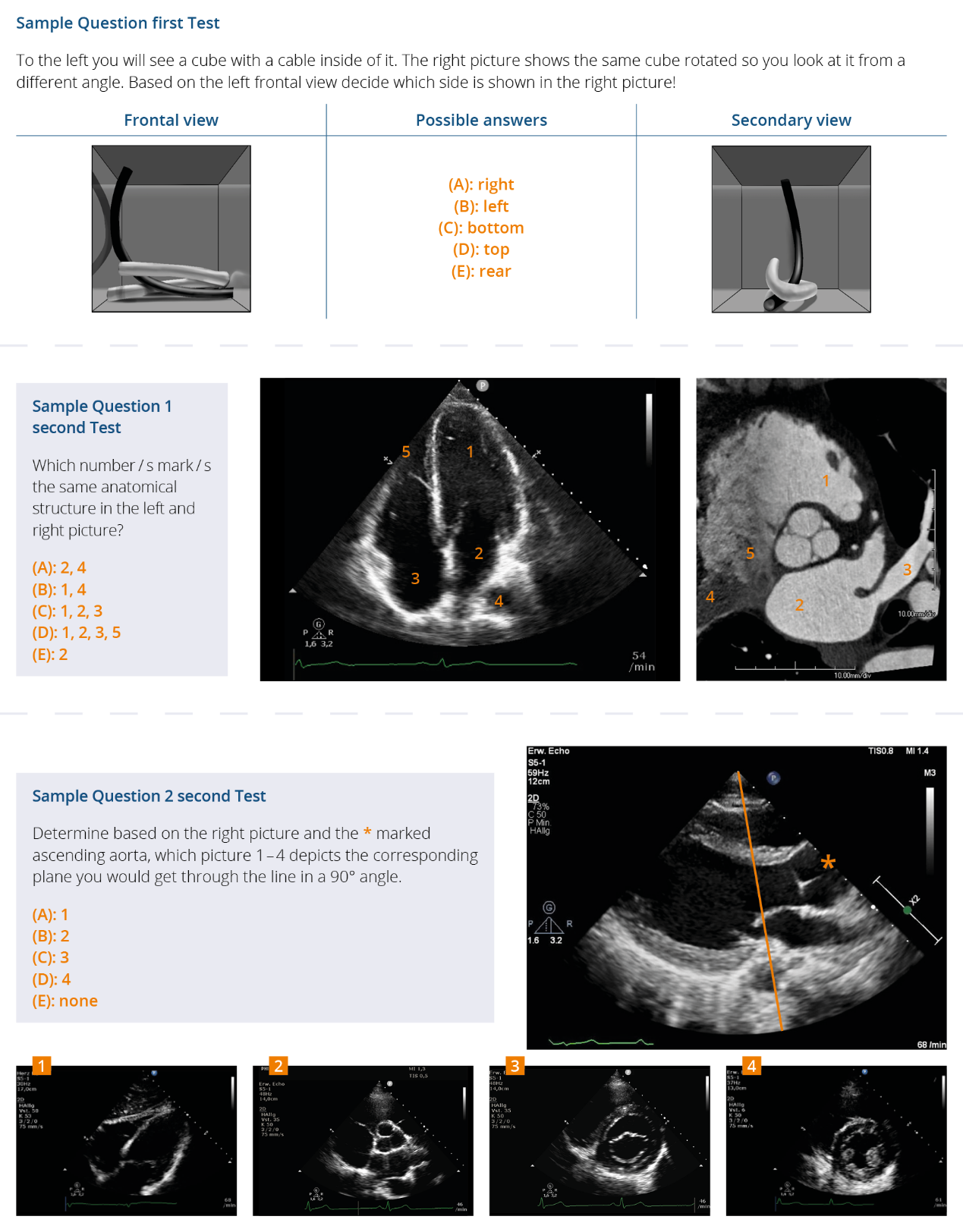

Supplement: Supplementary file 1 — Additional file 1: Supplement 1. Course sequence, adapted from Greim et al. [37]. Supplement 2. Rotation plan for the practice group. MAPSE, mitral annular plane systolic excursion; TAPSE, tricuspid annular plane systolic excursion; EPSS, mitral valve E-point to septal separation. Supplement 3. Excerpt from the test sections on “figural intelligence” and “technical knowledge”. [file 12909_2023_4062_MOESM1_ESM.docx]
